# P-345. The Changing Dynamics of the *Candida auris* Outbreak in Southern California

**DOI:** 10.1093/ofid/ofae631.547

**Published:** 2025-01-29

**Authors:** Bennett M Shaw, Daniel Uslan, Shangxin Yang

**Affiliations:** David Geffen School of Medicine at UCLA, Los Angeles, California; Division of Infectious Disease, UCLA Medical Center, LA, California; Univeristy of California, Los Angeles, Los Angeles, California, USA, Los Angeles, California

## Abstract

**Background:**

The current global outbreak of *Candida auris* poses a serious public health threat. With both high levels of antifungal resistance and a propensity for nosocomial spread, *Candida auris* has the potential to cause devastating outbreaks within healthcare facilities, especially amongst long-term and intensive care patients. As in many viral and bacterial pathogens, pathogen genomics has proven to be an indispensable piece of the outbreak response toolkit by contributing information useful in diagnosis, drug-resistance testing, and epidemiologic investigations.

Phylogenetic analysis of C. auris reveals introduction of Clade I to Southern California

**Figure 1.**
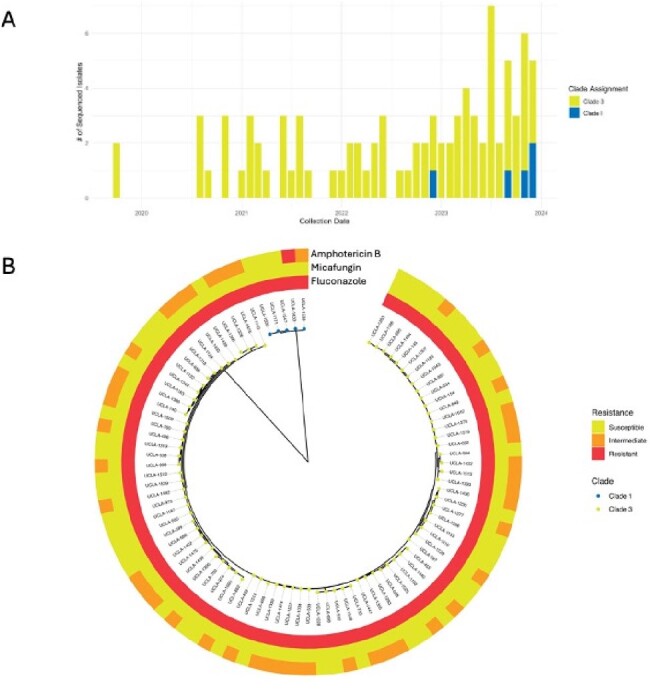
A) Distribution of sequenced C. auris case clade membership during study period. B) Maximum-likelihood phylogenetic tree constructed from concatenated SNP sites of sequenced C. auris isolates with antifungal resistance data. MIC breakpoints: Fluconazole ≥ 32, Micafungin ≥ 4, Amphotericin B Intermediate ≥ 2, Resistant ≥ 4

**Methods:**

At our large healthcare system located in Los Angeles, patients admitted with epidemiologic risk factors such as residence in a long-term care facility undergo admission screening for *C. auris* via combined axillary/inguinal swab. Microbiologic data from 213 *C. auris* isolates collected either via admission screening or from clinical cultures, including 91 isolates with paired whole-genome sequencing data, were used to conduct an in-depth investigation of the changing dynamics of the local *C. auris* outbreak. Phylogenetic analysis was employed to investigate the relationships between isolates.

**Results:**

Transmission of *C. auris* between 2021 and 2023 remained high, with an average of 59 patients per year determined to be colonized or infected with *C. auris*. While the local outbreak has overall been dominated by Clade III isolates that are resistant to fluconazole but susceptible to echinocandins and amphotericin B, phylogenetic analysis identified the first known introduction of Clade I to Southern California in late 2022, which may have led to the first amphotericin B resistant case within our healthcare system. Genotypes of *ERG11* and *FKS1* genes were found to correlate with fluconazole resistance and echinocandin susceptibility. Two cases identified nearly one month apart in an intensive care unit were genetically identical, supporting epidemiologic suspicion of in-hospital transmission.

**Conclusion:**

Continued genomic surveillance of *C. auris* contributed to an enhanced understanding of local outbreak dynamics, helping inform and guide empirical treatments and epidemiologic investigations.

**Disclosures:**

**All Authors**: No reported disclosures

